# A randomised multicentre phase II trial of capecitabine *vs* S-1 as first-line treatment in elderly patients with metastatic or recurrent unresectable gastric cancer

**DOI:** 10.1038/sj.bjc.6604536

**Published:** 2008-07-29

**Authors:** J-L Lee, Y-K Kang, H J Kang, K-H Lee, D Y Zang, B-Y Ryoo, J G Kim, S R Park, W K Kang, D B Shin, M-H Ryu, H M Chang, T-W Kim, J H Baek, Y J Min

**Affiliations:** 1 Department of Internal Medicine, Asan Medical Center, University of Ulsan College of Medicine, Seoul 138-736, Korea; 2 Korea Institute of Radiological and Medical Science, Seoul 139-706, Korea; 3 Yeungnam University Medical Center, Yeungnam University School of Medicine, Daegu 705-030, Korea; 4 Hallym University Medical Center, Hallym University College of Medicine, Anyang 431-070, Korea; 5 Kyungpook National University Hospital, Kyungpook National University College of Medicine, Daegu 700-721, Korea; 6 National Cancer Institute, Goyang 410-769, Korea; 7 Samsung Medical Center, Sungkyunkwan University School of Medicine, Seoul 135-710, Korea; 8 Gacheon Medical School Gil Medical Center, Gacheon University of Medical Science, Incheon 405-760, Korea; 9 Ulsan University Hospital, University of Ulsan College of Medicine, Ulsan 682-060, Korea

**Keywords:** capecitabine, S-1, gastric cancer, elderly, efficacy, safety

## Abstract

This randomised multicentre phase II study was conducted to investigate the activity and safety of two oral fluoropyrimidines, capecitabine or S-1, in elderly patients with advanced gastric cancer (AGC). Elderly (⩾65 years) chemo-naive patients with AGC were randomly assigned to receive capecitabine 1250 mg m^−2^ two times daily on days 1–14 every 3 weeks or S-1 40–60 mg two times daily according to body surface area on days 1–28 every 6 weeks. Ninety-six patients were enrolled and 91 patients were randomised to capecitabine (*N*=46) or S-1 (*N*=45). Overall response rate, the primary end point, was 27.2% (95% CI, 14.1–40.4, 12 of 44 assessable patients) with capecitabine and 28.9% (95% CI, 15.6–42.1, 13 of 45) with S-1. Median times to progression and overall survival in the capecitabine arm (4.7 and 9.5 months, respectively) were similar to those in the S-1 arm (4.2 and 8.2 months, respectively). The incidence of grade 3–4 granulocytopenia was 6.8% with capecitabine and 4.8% with S-1. Grade 3–4 nonhaematologic toxicities were: asthenia (9.1% with capecitabine *vs* 7.1% with S-1), anorexia (6.8 *vs* 9.5%), diarrhoea (2.3 *vs* 0%), and hand–foot syndrome (6.8 *vs* 0%). Both capecitabine and S-1 monotherapies were active and tolerable as first-line treatment for elderly patients with AGC.

Stomach cancer is the fourth most frequent malignant disease and the second most common cause of cancer-related deaths in the world (Parkin *et al*, 2005; Ries *et al*, 2007). Although the number of deaths from gastric cancer has declined over the past decades as a result of screening endoscopy, the elderly are primarily affected by the disease with most gastric cancer-related deaths occurring in patients aged 65 years or older (Ries *et al*, 2007).

Systemic chemotherapy improves the quantity and quality of life in patients with gastric cancer when compared with best supportive care alone (Murad *et al*, 1993; Pyrhonen *et al*, 1995; Glimelius *et al*, 1997). Various chemotherapeutic agents are used for the treatment of advanced gastric cancer (AGC). Some are used as single agents, others as part of combination regimens. 5-Fluorouracil (5-FU) has been the backbone of most regimens for AGC for several decades, and is used most commonly in combination with a platinum with or without an anthracycline or taxane (Kim *et al*, 1993; Webb *et al*, 1997; Waters *et al*, 1999; Vanhoefer *et al*, 2000; Van Cutsem *et al*, 2006). However, elderly cancer patients often present with concomitant co-morbidities and age-associated physiologic problems, such as impaired organ function and functional changes that make the selection of optimal treatment difficult (Repetto and Balducci, 2002; Lichtman *et al*, 2007b). There is also uncertainty about the use of systemic palliative chemotherapy in elderly patients because of under-representation of this age group in clinical trials (Murthy *et al*, 2002; Lewis *et al*, 2003; Lichtman *et al*, 2007a).

Capecitabine is an oral fluoropyrimidine carbamate, which is enzymatically converted to 5-FU in several steps following absorption from the gastrointestinal tract. The final step involves the enzyme thymidine phosphorylase, which is found at much higher levels in gastric cancers than in normal tissue, enabling the active drug 5-FU to be generated preferentially at the tumour site (Miwa *et al*, 1998). The efficacy and safety of the standard 3-weekly intermittent regimen of capecitabine for AGC has been demonstrated in a Korean study including 55 chemo-naive patients (Hong *et al*, 2004). The overall response rate (ORR) was 34% with a median overall survival (OS) of 9.5 months with a favourable safety profile. More recently, two randomised phase III trials in patients with AGC have been completed. The first multinational study showed that capecitabine/cisplatin was noninferior to 5-FU/cisplatin in terms of progression-free survival (Kang *et al*, 2006). The second trial (REAL 2), which was performed in the UK and Australia, demonstrated that capecitabine can replace 5-FU and oxaliplatin can replace cisplatin in triple combinations used for the treatment of advanced esophagogastric cancer (Cunningham *et al*, 2008). Capecitabine has since received approval for use in AGC on the basis of these trials.

S-1 is a novel oral fluoropyrimidine consisting of a 5-FU prodrug, tegafur, and the dihydropyrimidine dehydrogenase inhibitor, 5-chloro-2,4-dihydroxypyridine and the orotate phosphoribosyl transferase inhibitor, potassium oxonate, which suppresses the gastrointestinal toxicity of tegafur (Maehara, 2003). In two phase II studies in patients with AGC conducted in Japan, 80 mg m^−2^ of S-1 daily for 4 weeks followed by a 2-week rest period showed high ORRs of 49% (25 of 51) and 44% (19 of 43), respectively, with median OS of 8.3 and 6.8 months, respectively (Sakata *et al*, 1998; Koizumi *et al*, 2000). By virtue of their oral formulations, promising efficacy, and favourable toxicity profiles, capecitabine and S-1 may be particularly attractive for elderly cancer patients.

This phase II trial (NCT00278863) was conducted to evaluate the efficacy, safety, and feasibility of capecitabine or S-1 in elderly patients with previously untreated AGC and to assess the relative advantages of these agents in the treatment of AGC.

## Materials and methods

### Patients

Elderly patients (aged⩾65 years) with advanced, unresectable, histologically confirmed adenocarcinoma of the stomach or gastroesophageal junction were eligible if they met the following inclusion criteria: ECOG performance status 0–2; measurable disease based on RECIST criteria; no previous chemotherapy except adjuvant chemotherapy completed at least 12 months before enrollment; an estimated life expectancy of more than 3 months; ability for adequate oral intake; adequate bone marrow function, defined as leukocyte count ⩾4000 *μ*l^−1^, absolute neutrophil count (ANC) ⩾1500 *μ*l^−1^, haemoglobin ⩾9.0 g dl^−1^, and platelets ⩾100 000 *μ*l^−1^; adequate renal and hepatic function, defined as serum creatinine <1.5 mg dl^−1^, bilirubin <1.5 mg dl^−1^, and serum transaminase <3 times the upper normal limit (UNL) (<5 times UNL for patients with liver metastases); and written informed consent. Patients were excluded if they had brain metastases, significant gastrointestinal bleeding, a serious co-morbid condition, concomitant use of any drugs, which had a potential interaction with S-1 (flucytosine, allopurinol, warfarin, and phenytoin), or inability to comply with the requirements of the protocol. This study was initially approved by the Institutional Review Board (IRB) of the Asan Medical Center and later by all IRBs responsible for the participating centres.

### Study design and randomisation

This study was an open-label, multicentre, randomised phase II trial with two treatment arms. Random assignment (1 : 1 ratio) was centralised and performed by the Coordination Center for Clinical Trials on Gastrointestinal Cancer at the Asan Medical Center, Seoul, Korea. Randomisation was stratified by age (⩽75 years *vs* >75 years), performance status (0–1 *vs* 2), and prior gastrectomy (yes *vs* no).

### Treatment dose and schedule

Patients were randomly assigned to receive either capecitabine or S-1 as recommended in the package insert. Capecitabine 2500 mg m^−2^ was administered orally in two divided doses daily on days 1–14 of a 21-day cycle. The dosage of capecitabine was adjusted or interrupted for treatment-related adverse events of grade 2 or worse based on a previously defined algorithm (Blum *et al*, 1999). S-1 was given orally two times daily for 28 days, followed by 14 days’ rest. Three dosage levels of S-1 were defined according to body surface area (BSA) as follows: BSA less than 1.25 m^2^, 40 mg two times daily; BSA, 1.25 to 1.5 m^2^, 50 mg two times daily; and BSA more than 1.5 m^2^, 60 mg two times daily. S-1 was temporarily discontinued and the same dose was retried if patients experienced grade 2 nonhaematologic toxicities, grade 3 thrombocytopenia, or uncomplicated grade 4 neutropenia. If the toxicity recurred or grade 3 nonhaematologic toxicities, grade 4 thrombocytopenia, or febrile neutropenia occurred, S-1 was interrupted until the toxicity subsided to grade 1 or less. The BSA-adjusted S-1 dose was then reduced from 120 to 100 mg day^−1^, from 100 to 80 mg day^−1^, or from 80 to 50 mg day^−1^. The subsequent chemotherapy cycle was started only if the ANC recovered to ⩾1500 *μ*l^−1^ and the platelet count reached ⩾100 000 *μ*l^−1^, and nonhaematologic toxicity recovered to grade 1 or less. A treatment delay of up to 3 weeks was permitted without dose reduction. If the ANC was ⩾1000 *μ*l^−1^ but <1500 *μ*l^−1^ and the platelet count was ⩾75 000 *μ*l^−1^ but <100 000 *μ*l^−1^ on the scheduled day 1 of the next cycle after a 1-week delay, treatment could be started with a 25% reduced dose of capecitabine or at the next lowest dose level for S-1. Each treatment was continued until the occurrence of disease progression, unacceptable toxicities, or patient's refusal to continue.

### Pretreatment and on-treatment evaluation

No more than 2 weeks before study entry, patients underwent the following evaluations: medical history; complete physical examination including ECOG performance status; complete blood count, serum chemistry with electrolyte and coagulation battery; urinalysis; chest X-ray; electrocardiogram; and computed tomography (CT) of the abdomen and pelvis (CT of chest or neck was also performed if indicated). Charlson comorbidity index (CCI) was prospectively calculated before treatment (Charlson *et al*, 1987). Other investigations, for example, bone scan or bone X-ray, were performed if clinically indicated to document metastatic disease.

All patients were reviewed before the commencement of each cycle of chemotherapy. Complete and differential blood counts and serum chemistry were performed before each 21-day cycle for patients receiving capecitabine and before each 42-day cycle for patients receiving S-1. More frequent reviews and monitoring were undertaken if clinically indicated.

Compliance with study medications was monitored by questioning patients and counting their remaining pills at each visit. The ratio of the actual dose taken to the prescribed dose was calculated and used to calculate relative dose intensity.

All patients completed a quality-of-life (QOL) questionnaire (EORTC QLQ-C30, QLQ-STO22 Korean version) within 14 days before registration, with each even-numbered chemotherapy cycle for patients receiving capecitabine or each chemotherapy cycle for patients receiving S-1, at the end of the study treatment, and every 2 months thereafter. Results on QOL are to be reported in a separate publication.

### Response and toxicity criteria

Tumour response was assessed every two cycles in the capecitabine arm and every cycle for S-1 according to the RECIST criteria. At each of these assessments, the same imaging technique was used as was employed at baseline. National Cancer Institute Common Toxicity Criteria, version 2.0, were used to assess toxicity. Radiographs of all eligible patients were also reviewed extramurally to confirm investigator-designated responses.

### Statistical analysis

The primary end point was ORR as assessed by external independent review, which was analysed on both an intention-to-treat (ITT, all eligible patients who were randomly assigned) and per-protocol (PP, treated patients eligible and assessable for response without major protocol violations) basis. Patients were considered assessable for response if they had received at least two cycles of chemotherapy in the capecitabine arm and one cycle in the S-1 arm and had had at least one follow-up tumour assessment. However, patients were also considered assessable if they had received less than the predefined number of cycle(s) of chemotherapy due to rapid tumour progression.

Simon's optimal two-stage design was used for both treatment arms to allow early termination of inactive arm(s). To test the null hypothesis *P*_0_⩽0.1 *vs* the alternative hypothesis *P*_1_⩾0.25, the first stage required at least three or more patients out of 18 to have a confirmed response with *α*=0.05 and *β*=0.2 before proceeding to the second stage. In the second stage, 25 assessable patients could be added and if a total of eight or more patients achieved a confirmed response, then the primary end point would have been met.

The secondary end points were time-to-progression (TTP), time-to-treatment failure (TTF, including progression, death, or withdrawal), and OS. Kaplan–Meier estimates and the Cox proportional hazard model were used in the analysis of time-event variables. Comparison of the distribution of discrete variables in the two treatment arms were performed by the χ^2^ test or Fisher's exact test when appropriate, and further evaluated by logistic regression analysis. For continuous variables, the Mann–Whitney *U*-test for nonparametric data was used. All tests were two sided and a *P*-value<0.05 was considered as statistically significant. SPSS for Windows version 13.0 (SPSS Inc., Chicago, IL, USA) was used for statistical analyses.

## Results

### Patient characteristics

**Figure 1:**
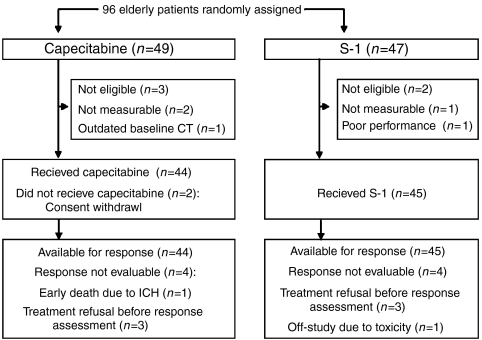
CONSORT diagram.

From October 2004 to April 2006, 96 patients from nine centres in Korea were enrolled; 49 were randomly assigned to receive capecitabine and 47 to S-1. As shown in Figure 1, five patients were ineligible and were excluded from the analysis. Therefore, the ITT population contained 91 patients. Two patients in the capecitabine arm withdrew consent without study treatment and were excluded from the PP analysis. The baseline characteristics were well balanced between the two treatment arms (Table 1). The median age of patients was 71 years (range, 65–82 years) and the CCI was 1 or more in 37% of patients.

### Exposure to study medication

**Figure 2:**
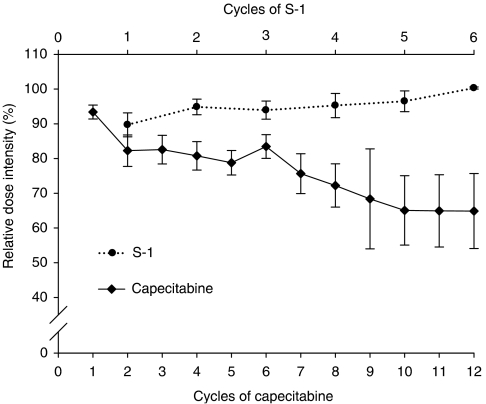
Relative dose intensity (median and standard error) for capecitabine (solid-line) and S-1 (dotted-line) over treatment cycles.

In total, 240 cycles of capecitabine and 144 cycles of S-1 were administered, with a median of five cycles (range, 1–22) in the capecitabine arm and two cycles (range, 1–14) in the S-1 arm. Thirty-four patients in the S-1 arm received 60 mg two times daily and the remaining 11 patients received 50 mg two times daily. The median relative dose intensity per patient was 87.6% (range, 32.3–102.4%) for capecitabine and 96.3% (range, 16.1–102.5%) for S-1 (*P*=0.003). Although the relative dose intensity for S-1 remained stable, there was a steady decrease in relative dose intensity for capecitabine during treatment (Figure 2). There were more dose reductions (27.5% of cycles and 36.4% of patients) and dose delays (12.7% of cycles and 31.8% of patients) with capecitabine than with S-1 (2.1% of cycles and 11.4% of patients for dose reductions, and 7.6% of cycles and 13.3% of patients for dose delays). Hand–foot syndrome (16.9% of cycles) and neutropenia (4.7% of cycles) were the most frequent reasons for dose reductions in the capecitabine arm. These toxicities did not result in dose reduction in the S-1 arm.

The main reason for discontinuing study treatment was disease progression (64% of patients in the capecitabine arm and 71% of patients in the S-1 arm). Other reasons included patient refusal (22.7% for capecitabine and 13.3% for S-1), death (4.5 and 4.4%, respectively), and other concomitant illnesses not associated with study treatment (6.8 and 6.6%, respectively). Only one patient in each treatment group was taken off study treatment because of intolerable treatment-related toxicities: recurrent grade 2 vomiting in the capecitabine arm, and grade 3 abdominal pain in the S-1 arm.

### Treatment response

The ORR for both the ITT and PP populations are shown in Table 2. Both treatments met the primary end point of this study. The confirmed ORR for the ITT population was 26.1% for capecitabine (95% CI, 13.4–38.8) and 28.9% for S-1 (95% CI, 15.6–42.1). ORR for the PP population was 27.2% for capecitabine (95% CI, 14.1–40.4) and 28.9% for S-1 (95% CI, 15.6–42.1). The median duration of response in the ITT population was 6.3 months for capecitabine (95% CI, 5.46–7.14) and 8.5 months for S-1 (95% CI, 5.70–11.26), with a hazard ratio (capecitabine/S-1) of 1.3 (95% CI, 0.54–3.20). The reason that patients were not assessed for efficacy are shown in Figure 1. Logistic regression analysis revealed that none of the potential prognostic factors were statistically significant predictive factors for overall response (Table 3).

### Secondary end points

At the last data cutoff date, 15 May 2007, the median follow-up duration was 21.9 months in the capecitabine arm (95% CI, 19.4–24.5) and 21.7 months in the S-1 arm (95% CI, 18.4–25.0). At this time, 73 of the 91 patients had died. Disease progression was the major cause of death and accounted for 95.8% of patient deaths in both treatment groups. Two patients in the capecitabine arm died from non-cancer-related causes (intracranial haemorrhage, *n*=1; sudden cardiac death, *n*=1) both of which had no direct causal relationship with study medication. The cause of death in one patient allocated to S-1 was unclear.

**Figure 3:**
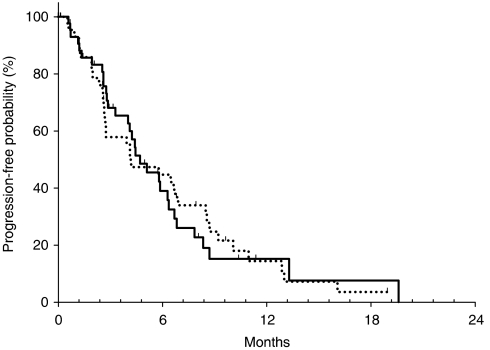
Time-to-progression curves for patients with advanced gastric cancer treated with capecitabine (solid-line) and S-1 (dotted-line).

**Figure 4:**
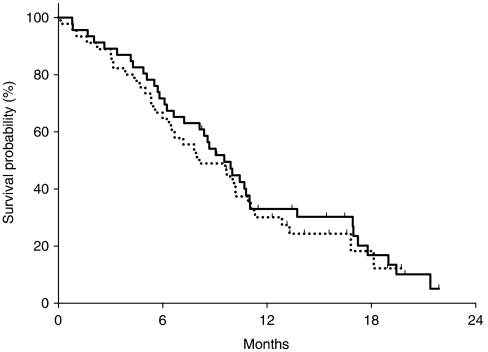
Overall survival curves for patients with advanced gastric cancer treated with capecitabine (solid-line) and S-1 (dotted-line).

The median TTP was 4.7 months for capecitabine (95% CI, 3.1–6.4) and 4.2 months for S-1 (95% CI, 1.5–6.9), with a hazard ratio (capecitabine/S-1) of 1.0 (95% CI, 0.6–1.6) (Figure 3). The probability of remaining progression-free at 6 months was 35.8 and 42.2% for the capecitabine and S-1 arms, respectively. The median TTF was 4.3 months for capecitabine (95% CI, 2.8–5.7) and 3.0 months for S-1 (95% CI, 1.5–4.5). The median OS was 9.5 months for capecitabine (95% CI, 7.8–11.3) and 8.1 months for S-1 (95% CI, 4.9–11.4); the hazard ratio (capecitabine/S-1) was 0.9 (95% CI, 0.5–1.4) (Figure 4). The 1-year survival rate was 30.2% for capecitabine and 27.3% for S-1. Cox proportional hazard analyses found that none of the potential prognostic factors were statistically significant predictive factors for OS (Table 3). However, patients who received prior gastrectomy had tendency towards a longer OS according to both univariate and multivariate analyses.

### Safety

Haematologic and nonhaematologic adverse events are shown in Table 4. The incidence of severe adverse events was very low in both treatment groups. With the exception of hand–foot syndrome and stomatitis, which were more frequently found in the capecitabine arm, the incidence of grade 2 or worse toxicities was similar in the two treatment arms.

## Discussion

This is the first randomised trial of two new oral fluoropyrimidines, capecitabine or S-1, in elderly patients with AGC. Although this was a phase II study and did not aim to compare the treatment groups statistically, we could estimate the relative efficacy and safety of the two drugs. Elderly patients may be the best target population for this purpose because single-agent chemotherapy can be a good and safe first-line treatment option for this patient group and it avoids the compounding effects of other agents if combination therapy is used. For the assessment of the primary study end point, ORR, all images were extramurally reviewed and all responses were confirmed. Both agents met the primary end point and seemed to have similar activity in elderly patients with AGC. In the PP population, ORRs of 28.9% with S-1 and 27.2% with capecitabine were observed. The ORR for capecitabine is largely consistent with a previous study performed in patients with AGC (Hong *et al*, 2004), whereas that for S-1 is somewhat lower than that observed in Japanese trials (Sakata *et al*, 1998; Koizumi *et al*, 2000), but is better than that observed in a non-Japanese Asian study (Jeung *et al*, 2007). Median TTF, TTP and OS were also very similar for both regimens.

Capecitabine and S-1 were well tolerated and no treatment-related deaths were reported in this study. Both agents were minimally myelosuppressive and the most frequent haematological toxicity was anaemia. Although one episode of febrile neutropenia occurred in one patient treated with capecitabine, there were no meaningful differences in haematologic toxicities between the two treatment arms. The most frequently observed grade 3/4 nonhaematological toxicities were anorexia and asthenia. Although these toxicities developed in less than 10% of patients, they had a tendency to become more clinically significant as treatment progressed. The only notable differences in grade 2 or worse nonhaematologic adverse events between S-1 and capecitabine were the greater incidences of hand–foot syndrome and stomatitis among patients treated with capecitabine. There was a steady decrease in the relative dose intensity of capecitabine during treatment caused by treatment delays or dose reductions mostly because of hand–foot syndrome, whereas that of S-1 remained steady (Figure 2). Considering these findings, we suggest that a dose of 1000 mg m^−2^ two times daily, lower than that recommended, would be appropriate for capecitabine in elderly patients (Bajetta *et al*, 2005), whereas the recommended dose of 40 mg m^−2^ two times daily would be acceptable for S-1. These findings suggest that dose escalation of S-1 may be possible in younger patients.

The rates of some key adverse events reported with capecitabine in our study, as well as in other phase II studies performed in Japan or Korea (Hong *et al*, 2004; Lee *et al*, 2004; Hyodo *et al*, 2006), appear to differ from those reported in phase III multinational studies (Cassidy *et al*, 2002; Twelves *et al*, 2005). The rates of grade 3 hand–foot syndrome (6–13% in Asian phase II studies *vs* 17% in multinational phase III studies) and grade 3/4 diarrhoea (2–5 *vs* 11–13%) tend to be lower, and the rate of grade 3/4 neutropenia (0–8 *vs* 1–2%) similar or higher. These observations are consistent with the recent work of Haller *et al* (2008) who reported regional differences in the tolerability of fluoropyrimidines. They found that the risk of adverse events was lowest in patients from East Asian centres, except for grade 3/4 neutropenia, which was most likely to occur in patients from East Asia. No safety data regarding S-1 monotherapy from the United States or Europe are yet available.

According to recent SEER data from the United States, 65.5% of gastric cancers are diagnosed in patients older than 65 years: the median age at diagnosis of gastric cancer was 71 years and the median age of gastric cancer-related death was 74 years (Ries *et al*, 2007). However, because elderly patients are generally excluded from cancer chemotherapy clinical trials, data to guide the treatment of older patients with AGC in an evidence-based fashion are lacking (Lewis *et al*, 2003; Murthy *et al*, 2004; Trumper *et al*, 2006; Lichtman *et al*, 2007b). The recently reported SPIRITS trial and a meta-analysis on first-line chemotherapy in patients with AGC demonstrated a statistically significant benefit in OS for 5-FU-based combination chemotherapy compared with single-agent chemotherapy (Wagner *et al*, 2006; Koizumi *et al*, 2008). Trumper *et al* (2006) suggested that elderly (⩾70 years) patients without significant co-morbidities should be treated with the same regimens as younger patients with AGC based on a retrospective review of three UK multicentre randomised trials. However, the SPIRITS trial only involved patients of less than 75 years, and extrapolation of the results from retrospective reviews or meta-analyses to the elderly patients must be done with caution because of the following limitations: (1) a small, approximately 1-month, survival advantage observed in the meta-analysis was achieved at the expense of increased toxicity (Wagner *et al*, 2006); (2) chemotherapy-related toxicities, such as neutropenia, anaemia, stomatitis and diarrhoea, occurred more frequently in the elderly (Trumper *et al*, 2006); (3) the early drop out rate was significantly higher and 5-FU dose intensity was significantly lower in the elderly when treated with combination chemotherapy containing 5-FU and cisplatin (Trumper *et al*, 2006); and (4) QOL, which could be impaired as the intensity of chemotherapy increases, has not been studied sufficiently. Ideally, standard treatment of AGC in elderly patients should not be based solely on retrospective subset analyses of prospective trials, and elderly specific trials are needed to define the optimal treatment for these patients (Perrone *et al*, 2002; Jatoi *et al*, 2005). Considering the ORR, OS and safety results, our study provides evidence that elderly patients with AGC could benefit from capecitabine or S-1 monotherapy with minimal adverse events.

In conclusion, our study indicates that both capecitabine and S-1 are safe, well tolerated and efficacious in older patients with AGC. Oral agents, such as capecitabine or S-1, may have particular appeal in the management of cancer in this patient population. An additional trial is needed to clarify the potential predictive factors for drug selection and to establish the effectiveness of various combinations, including molecular targeted agents, in older patients with AGC.

## Figures and Tables

**Table 1:** Patient characteristics at baseline (ITT population)

	**Capecitabine**	**S-1**
	**No. (%)**	**No. (%)**
No. of patients	46	45
		
*Age (years)*		
Median	71	71
Range	66–78	65–82
		
*ECOG performance status*		
0	7 (15.2)	10 (22.2)
1	35 (76.1)	33 (73.3)
2	4 (8.7)	2 (4.4)
		
*Gender*		
Male	30 (65.2)	37 (82.2)
Female	16 (34.8)	8 (17.8)
		
*Body surface area (m^2^)*		
Median	1.50	1.60
Range	1.24–1.91	1.26–2.05
		
*Charlson comorbidity index*		
0	28 (60.9)	29 (64.4)
1	17 (40.0)	12 (26.7)
2	0	3 (6.7)
3–4	1 (2.2)	1 (2.2)
Prior gastrectomy	10 (21.7)	10 (22.2)
Adjuvant chemotherapy	2 (4.3)	4 (8.9)
Elevated alkaline phosphatase	11 (23.9)	12 (26.7)
		
*Metastatic sites*		
Liver	28 (60.9)	22 (48.9)
Peritoneum	8 (17.4)	13 (28.9)
Bone	1 (2.2)	0
Lymph node	31 (67.4)	29 (64.4)
		
*No. of metastatic organs*		
1	23 (50.0)	24 (53.3)
2	17 (37.0)	13 (28.9)
⩾3	6 (13.0)	8 (17.8)

**Table 2:** Response rates according to RECIST criteria (ITT and PP populations)

	**ITT population**				**PP population**			
	**Capecitabine (*N*=46)**		**S-1 (*N*=45)**		**Capecitabine (*N*=44)**		**S-1 (*N*=45)**	
	**INV**	**EIR**	**INV**	**EIR**	**INV**	**EIR**	**INV**	**EIR**
*Response, n*								
CR	2	0	1	1	2	0	1	1
PR	11	12	11	12	11	12	11	12
SD	19	18	20	18	19	18	20	18
PD	8	10	9	10	8	10	9	10
NA	6	6	4	4	4	4	4	4
ORR, *n* (%)		12 (26.1)		13 (28.9)		12 (27.2)		13 (28.9)
95% CI		13.4–38.8		15.6–42.1		14.1–40.4		15.6–42.1

INV=investigator assessment; EIR=external independent review; ORR=overall response rate; CR=complete response; PR=partial response; SD=stable disease; PD=progressive disease; NA=not assessable; CI=confidence interval.

**Table 3:** Exploratory analysis of effects of prognostic factors on clinical outcome

		**Hazard ratio (95% confidence interval)**			
		**Overall response**		**Overall survival**	
**Factor**		**Univariate**	**Multivariate**	**Univariate**	**Multivariate**
Age, years	>70 *vs* ⩽70	1.4 (0.5–3.6)	1.3 (0.5–3.5)	1.2 (0.7–1.8)	1.2 (0.7–2.0)
ECOG performance status	⩾1 *vs* 0	1.1 (0.3–3.5)	0.82 (0.2–3.9)	1.4 (0.6–1.9)	1.3 (0.7–2.4)
Peritoneal involvement	Yes *vs* no	2.3 (0.8–6.6)	2.8 (0.9–8.5)	1.5 (0.9–2.6)	1.2 (0.6–2.1)
Liver involvement	Yes *vs* no	0.5 (0.2–1.3)	0.4 (0.2–1.3)	1.5 (1.0–2.5)	1.5 (0.9–2.6)
Charlson comorbidity index	⩾1 *vs* 0	0.9 (0.3–2.5)	1.0 (0.3–2.9)	1.3 (0.8–2.1)	1.1 (0.7–1.8)
Gastrectomy	Yes *vs* no	1.2 (0.4–3.3)	0.9 (0.3–2.8)	0.6^*^ (0.4–1.0)	0.6^*^ (0.3–1.0)
Elevated alkaline phosphatase	Yes *vs* no	1.2 (0.4–3.5)	1.7 (0.5–5.6)	1.0 (0.6–1.8)	0.9 (0.5–1.6)
Treatment	X *vs* S	0.9 (0.4–2.4)	1.4 (0.5–4.1)	1.0 (0.6–1.6)	0.9 (0.6–1.5)

^*^P=0.055 and *P*=0.07.

**Table 4:** Haematologic and nonhaematologic toxicities by severity

	**National Cancer Institute common toxicity criteria grade, no. of patients**											
	**Capecitabine (*N*=44)**						**S-1 (*N*=42)^a^**					
	**1**	**2**	**3**	**4**	**All grades (%)**	**3–4 (%)**	**1**	**2**	**3**	**4**	**All grades (%)**	**3–4 (%)**
Anaemia	19	15	3	2	39 (88.6)	5 (11.4)	15	16	3	3	37 (88.1)	6 (14.3)
Leukopenia	8	1	1	0	10 (22.7)	1 (2.3)	10	3	1	0	14 (33.3)	1 (2.4)
Granulocytopenia	8	2	2	1	13 (29.5)	3 (6.8)	5	3	2	0	10 (23.8)	2 (4.8)
Thrombocytopenia	10	1	1	0	12 (27.3)	1 (2.3)	9	2	0	1	12 (28.6)	1 (2.4)
Febrile neutropenia			1	0	1 (2.3)	1 (2.3)			0	0	0	0
Asthenia	24	6	4	0	34 (77.3)	4 (9.1)	26	5	3	0	34 (81.0)	3 (7.1)
Anorexia	28	7	3	0	38 (86.4)	3 (6.8)	19	8	4	0	31 (73.8)	4 (9.5)
Nausea	18	4	0	0	22 (50.0)	0	16	5	2	0	23 (54.8)	2 (4.8)
Vomiting	7	3	0	0	10 (22.7)	0	5	4	1	0	10 (23.8)	1 (2.4)
Abdominal pain	14	4	1	0	19 (43.2)	1 (2.3)	9	7	4	0	20 (47.6)	4 (9.5)
Stomatitis	15	9	0	0	24 (54.5)	0	6	3	0	0	9 (21.4)	0
Diarrhoea	11	4	1	0	16 (36.4)	1 (2.3)	11	2	0	0	13 (31.0)	0
Hand–foot syndrome	8	15	3	0	26 (59.1)	3 (6.8)	6	1	0	0	7 (16.7)	0
Transaminase	10	0	0	0	10 (22.7)	0	7	2	2	0	11 (26.2)	2 (4.8)
Hyperbilirubinaemia	10	4	0	1	15 (34.1)	1 (2.3)	9	4	0	0	13 (31.0)	0

a Toxicity assessment was not available in three patients who did not revisit the clinic after receiving the first cycle of chemotherapy.
